# Nutritional Strategies to Manage Malnutrition and Sarcopenia following Liver Transplantation: A Narrative Review

**DOI:** 10.3390/nu15040903

**Published:** 2023-02-10

**Authors:** Amal Trigui, Christopher F. Rose, Chantal Bémeur

**Affiliations:** 1 Department of Nutrition, Faculty of Medicine, Université de Montréal, Montreal, QC H3T 1A8, Canada; 2 Centre de Recherche du Centre Hospitalier de l’Université de Montréal (CRCHUM), Montreal, QC H2X 0A9, Canada; 3 Department of Medicine, Faculty of Medicine, Université de Montréal, Montreal, QC H3T 1J4, Canada

**Keywords:** liver transplant, malnutrition, sarcopenia, nutritional intervention, infections, hospital length of stay, acute cellular rejection, mortality

## Abstract

Persisting or newly developed malnutrition and sarcopenia after liver transplant (LT) are correlated with adverse health outcomes. This narrative review aims to examine the literature regarding nutrition strategies to manage malnutrition and sarcopenia after LT. The secondary aims are to provide an overview of the effect of nutrition strategies on the incidence of infections, hospital length of stay (LOS), acute cellular rejection (ACR), and mortality after LT. Four databases were searched. A total of 25 studies, mostly of mid–high quality, were included. Six studies found a beneficial effect on nutritional parameters using branched-chain amino acids (BCAA), immunomodulating diet (IMD), or enteral nutrition (EN) whereas two studies using beta-hydroxy-beta-methylbutyrate (HMB) found a beneficial effect on muscle mass and function. Fourteen studies using pre- or pro-biotics, IMD, and EN were effective in lowering infection and six studies using IMD, BCAA or HMB reported reduced hospital LOS. Finally, four studies using HMB and vitamin D were effective in reducing ACR and one study reported reduced mortality using vitamin D after LT. In conclusion, nutritional intervention after LT has different beneficial effects on malnutrition, sarcopenia, and other advert outcomes. Additional large and well-constructed RCTs using validated tools to assess nutritional status and sarcopenia are warranted to ensure more robust conclusions.

## 1. Introduction

The liver is the second most transplanted solid organ worldwide, representing 22% of all transplant procedures in 2021 [1]. Nevertheless, organ availability continues to be a major issue since the number of patients on the waiting list exceeds the number of livers available, rendering the waiting period for a deceased donor transplant between days to years [2]. The most common indication for LT is decompensated cirrhosis, the irreversible end-stage of chronic liver disease, characterized by the presence of complications such as malnutrition, hepatic encephalopathy, ascites, hepatocellular carcinoma, hepatorenal syndrome, or gastrointestinal bleeding caused by portal hypertension [3].

In patients with chronic liver disease awaiting LT, malnutrition is the most common complication, essentially due to a combination of inadequate dietary intake, malabsorption, and metabolic disturbances [4,5]. After LT, the replacement of the diseased liver with a functional liver leads to an improvement in nutritional deficiencies and metabolic disorders [6]. However, the patients’ nutritional status can worsen rapidly in the early postoperative period due mainly to perioperative malnutrition, surgical stress, immunosuppressive therapy, postoperative protein catabolism, and fasting periods [7,8,9]. In the immediate phase after LT, patients are hypercatabolic, as evidenced by the excretion of large amounts of urinary nitrogen, when compared with their state before LT [10]. Importantly, when the patient is malnourished before LT, the stress and inflammatory response due to surgery is prolonged [9]. Immunosuppressive agents, used after the surgery, are known to exert metabolic effects, which may be implicated in nutritional changes and body composition modifications [11]. Accordingly, corticosteroids increase appetite and fat deposition and decrease fat oxidation [12] whereas calcineurin inhibitors, such as cyclosporine and tacrolimus, affect energy metabolism [13,14]. In a large cohort study, malnutrition after LT was independently associated with early post-transplant morbidity such as infection, intensive care unit (ICU) and hospital LOS, which significantly increase hospital costs [15].

Malnutrition is one of the main risk factors for the onset and progression of sarcopenia [16,17]. The European Working Group on Sarcopenia in Older People (EWGSOP) defines sarcopenia as low muscle strength and low muscle mass or quality [17]. Sarcopenia may occur as a result of aging or chronic diseases, including cirrhosis [17,18]. Following LT, even though the metabolic complications of cirrhosis reverse, body composition does not always improve [19,20]. Nguyen et al. showed that 80% of cirrhotic patients were sarcopenic in the first 3 months after LT [21]. Of the patients who did not have sarcopenia pre-LT, 42% developed *de novo* sarcopenia postoperatively [22]. Immediately after surgery, Plank et al. reported a loss of 5 kg mainly from skeletal muscle which was not replenished up to twelve months thereafter [21]. Sarcopenia after LT is associated with poorer clinical outcomes such as graft rejection, longer hospital LOS, higher rates of infections, physical limitations, decreased quality of life, and mortality [22,23,24]. In addition to malnutrition, there are other factors causing sarcopenia after LT including postoperative hypercatabolism, development of posttransplant infections that worsen the catabolic state, hospitalization associated with physical inactivity, posttransplant renal failure, insulin resistance, and immunosuppressive therapy [25]. Immunosuppressive drugs such as corticosteroids, calcineurin inhibitors, and mammalian target of rapamycin (mTOR) inhibitors, extensively used post-LT, are all known to negatively affect the muscles [26,27]. Corticosteroids increase proteolysis and impair protein synthesis which leads to atrophy of muscle fibers [27]. Calcineurin inhibitors such as cyclosporine and tacrolimus, decrease muscle mitochondrial function, increase energy expenditure and metabolism, as well as impair muscle growth and regeneration [25,28,29]. Finally, mTOR inhibitors, such as sirolimus and everolimus, block muscle hypertrophy by inhibiting translation and protein synthesis [30].

Taken together, the presence of malnutrition and sarcopenia is strongly correlated with morbidity including infection and mortality after LT [18]. Decreased muscle mass after surgery has an impact on both rejection and infection [31]. The occurrence of infection after the surgery increases hospital LOS by more than 3 weeks [32]. Subsequently, hospitalization itself can impact exercise capacity and lead to functional decline after solid organ transplant which represents a component of sarcopenia [33]. For these reasons, nutritional support after LT is important for preventing and treating malnutrition and sarcopenia that could persist or newly appear after LT. The European Society for Parenteral and Enteral Nutrition (ESPEN) guidelines recommend early initiation of oral food intake or EN after organ transplantation, including energy intake of 35–40 kcal/kg/day and protein intake of 1.2–1.5 g/kg/day [9].

In this context, the overarching aim of this narrative review is to examine the recent literature regarding nutrition strategies that are effective to manage malnutrition and sarcopenia post-LT. Given their association with malnutrition and sarcopenia, our secondary aims are to provide an overview of the effect of nutrition strategies on the incidence of infections, hospital LOS, ACR, and mortality after LT as well as discuss tools used for the screening and diagnosis of malnutrition and sarcopenia.

## 2. Materials and Methods

### 2.1. Search Strategy

A literature search was carried out using Medline, Embase, and CINHAL complete to identify all nutritional intervention studies from inception to September 2022. Google Scholar was also used to identify additional articles by hand searching. The strategy utilized a combination of keywords and controlled vocabulary combining terms and synonyms related to post-LT AND nutrition intervention AND at least one of these outcomes: malnutrition or sarcopenia or infections or hospital LOS or ACR or mortality. The detailed strategy is as follows: “liver transplant” or “post-liver transplant*” or “hepatic transplant*” or “liver graft*” AND “after” or “preoperative” or “post*” or “following” or “postoperative” or “peri-operative” AND “BCAA” or “nutrition* intervention” or “branched-chain amino acids” or “beta-hydroxy, beta methylbutyrate” or “HMB” or “Beta hydroxy beta methylbutyrate” or “hydrolyzed whey peptide” or “immune*” or “immune* modulating” or “enteral nutrition” or “parenteral nutrition” or “probiotic*” or “prebiotic*” or “Synbiotic*” or “vitamin d” or “vitamin d3” or “cholecalciferol” or “supplement*” or “diet” or “ω-3 polyunsaturated fatty acids” or “omega-3 fatty acids” or “omega 3” or “fish oil” or “n-3” or “eicosapentaenoic” or “docosahexaenoic” or “glutamine” or “arginine” or “ribonucleic acids” or “immunonutrition” AND “malnutrition” or “nutritional deficiency” or “nutrients” or “nutritional markers” or “intake” or “sarcopenia” or “muscle mass” or “muscle function” or “muscle” or “muscle performance” or “physical performance” or “body composition” or “infections” or “infectiou*” or “hospital stay” or “length of hospital stay” or “hospital*” or “acute cellular rejection*” or “rejection*” or “mortality” or “survival rate”.

The final search was de-duplicated, and references were screened in Covidence. Titles and abstracts were checked to ensure that the target population was addressed and that they were indeed intervention studies. For potentially eligible articles, full texts were retrieved. After full-text review, the publications of interest were included.

### 2.2. Inclusion and Exclusion Criteria

The inclusion criteria were as follows: (1) adult females or males that received LT (2), all nutritional intervention including EN, parenteral nutrition (PN), oral supplementation, specific nutrient, or nutrient combination after LT, (3) studies reporting at least one of the following outcome measures: nutritional parameters, muscle mass, muscle function or performance, infection, hospital LOS, ACR, or mortality. The exclusion criteria were as follows: (1) nutritional intervention starting more than a week before LT, (2) studies involving the pediatric population, (3) reviews, (4) only abstract, and (5) articles published in a language other than English. There were no criteria based on geographic location of the studies.

### 2.3. Study Quality Assessment

The validated Mixed Methods Appraisal Tool (MMAT) was used to assess the methodological quality of the included studies [34]. This rigorous and easy-to-use tool was selected because it allows the simultaneous evaluation of studies of various research designs. An overall score was estimated for each study. This score ranges from 0% to 100%; 0% = very low quality, no criteria met; 20% = low quality, 1 criterion met; 40% = mid-low quality, 2 criteria met; 60% = mid-high quality, 3 criteria met; 80%= high quality, 4 criteria met; 100% = very high quality, all criteria met. Two reviewers (AT and CB) independently evaluated the quality of the articles included. Disagreements were resolved through discussion.

## 3. Results

### 3.1. Study Selection

**Figure 1:**
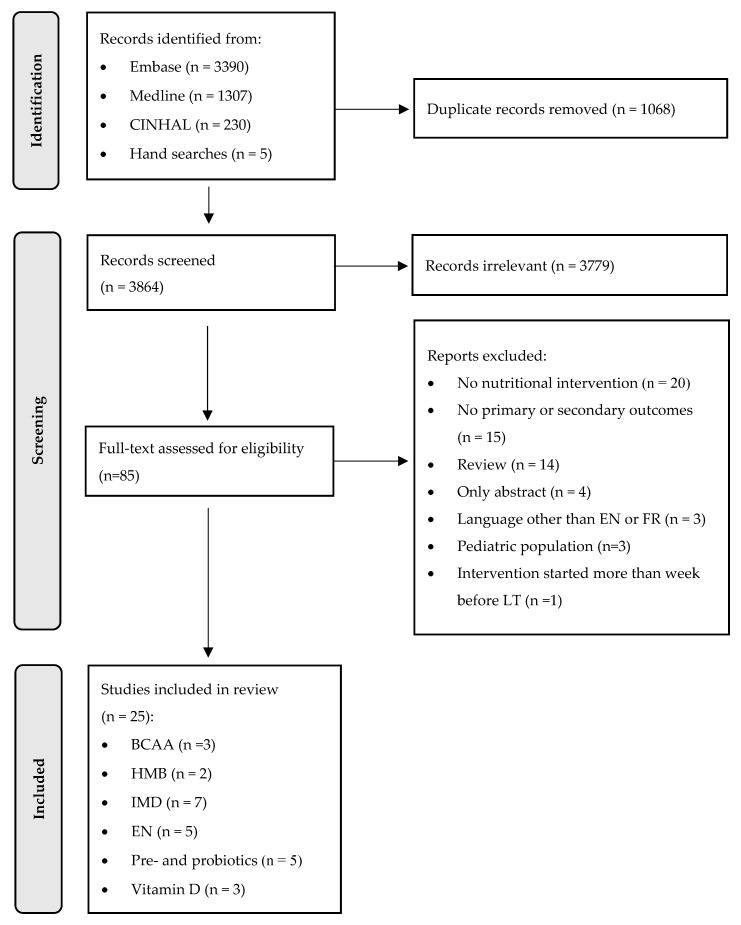
Flow diagram of study selection.

The flowchart illustrated in Figure 1 demonstrates the selection process of the articles. A total of 4 934 articles resulted in the initial search in the databases including 5 articles from hand searches. A total of 85 articles were retrieved. Sixty articles were excluded for not reporting on the inclusion and exclusion criteria. Finally, 25 studies were included in this review from which 16 were RCTs, 3 were non-randomized controlled trials, and 6 were retrospective intervention studies. Participant number ranges from 22 to 528. Intervention duration ranged between one and 12 weeks. The 25 articles included were divided into 6 tables according to the type of intervention: (1) BCAA, (2) HMB, (3) immune-modulating substances, (4) EN, (5) probiotics and prebiotics, and (6) vitamin D.

### 3.2. Study Quality

**Figure 2:**
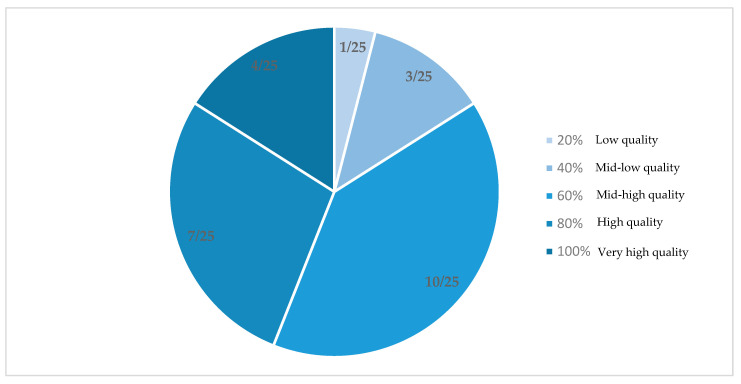
Study quality assessed by MMAT.

All studies underwent a quality assessment based on the MMAT. The results of these analyzes were presented in the 6 tables of each type of intervention and in Figure 2. The studies included in the review were mostly of mid-high quality, with a rating of 60% (10 articles). Only one study received a rating of 20% (low quality), three received a rating of 40% (mid-low quality), seven received a rating of 80% (high quality) whereas four were rated 100% (very high quality).

### 3.3. Nutritional Intervention Studies after Liver Transplant

#### 3.3.1. Branched-Chain Amino Acids (BCAA)

The three BCAA, leucine, isoleucine and valine, are among the nine essential amino acids [35]. Serum concentrations of BCAAs are decreased in patients with chronic liver diseases, while the concentrations of the aromatic amino acids (AAAs; phenylalanine, tryptophan, and tyrosine) are increased [36]. AAA levels return to normal after LT, whereas BCAA levels improve but are not normalized as they remain significantly lower compared to age-matched healthy individuals [37]. Interestingly, low levels of BCAAs are associated with low muscle mass, low strength, and poor muscle function in the elderly [38].

##### Effect of BCAA on Nutritional Parameters after LT

The effect of BCAAs on markers associated with nutritional status after LT has been investigated in three studies [39,40,41] (Table 1). In a prospective controlled study, Krapf et al. showed that patients following a nutritional program including ingredients naturally rich in BCAA and low in AAA for 14 days after LT, had a higher amount of food intake compared to the control group [39]. However, BCAAs levels were not significantly different between the groups [39]. The improvement in food intake could be the consequence of a specifically designed nutritional program in the intervention group. In another prospective randomized pilot study, Yoshida et al. demonstrated that early BCAA-enriched EN (12.15 g of BCAAs/day), which started 6 days before living donor liver transplantation (LDLT) and continued for 4 weeks after LDLT, improved serum biochemical nutritional parameters such as BCAA-to-tyrosine ratio (BTR) and retinol-binding protein [41]. In the same study, energy metabolism, assessed by the non-protein respiratory quotient, and rapid turnover proteins significantly increased in the BCAA group compared to the control group [41]. In the third study, Reilly et al. found the addition of BCAA within the PN after LT does not have any additional effect on nitrogen balance in patients with hypoalbuminemia before the surgery [40]. Both PN with and without BCAA were able to achieve the nitrogen balance immediately after LT. However, the BCAA to AAA ratio is increased in patients receiving supplemental BCAA [40]. Interestingly, an alteration in BCAAs and AAAs metabolism results in a low BTR which is an indicator of amino acid imbalance and a significant predictive factor for a decreased skeletal muscle mass [42]. Correcting the BCAA to AAA ratio or BTR after LT may have therapeutic potential for muscle mass and nutritional status improvements.

##### Effect of BCAA on Infections, Hospital Length of Stay and Mortality after LT

Lower BCAA levels are associated with an increase in postoperative infections [43]. Before LT, non-BCAA supplementation is an independent risk factor for bacteremia [44]. However, Yoshida et al. found no significant differences in the occurrence of infections between the BCAA group and the control group [41]. Furthermore, BCAA supplementation had no effect on hospital LOS and mortality after LT [39,40,41]. Only one study reported that BCAAs were well tolerated immediately after LT [40] (Table 1).

**Figure 3:**
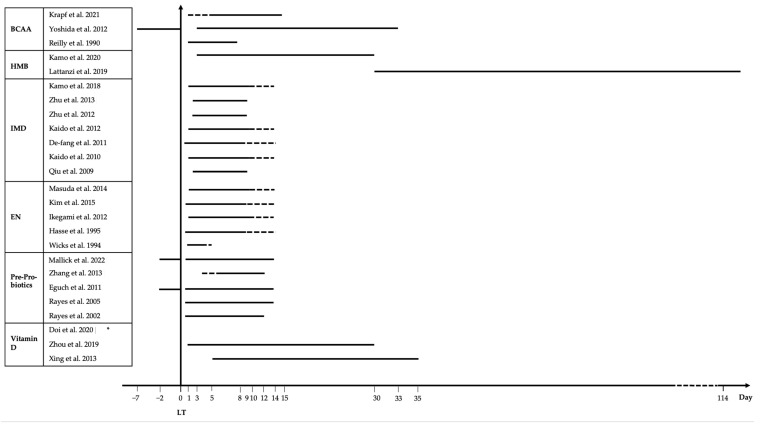
Duration of nutritional interventions studies after LT of included studies. Dotted lines at the beginning or at the end of the intervention indicates that the duration of the intervention differs between patients in the same study. * Duration varies between participants [39,40,41,46,51,57,61,64,65,66,67,69,70,71,74,75,76,84,87,88,89,90,91,100,101].

Based on these three studies, early post-LT BCAA supplementation showed beneficial effects on markers associated with nutritional status, energy metabolism, and protein turnover. However, no studies have assessed the effect of BCAAs on nutritional status using validated tools. In addition to providing energy, BCAAs build blocks for proteins and prevent muscle atrophy. Particularly, leucine promotes cell growth by overt nutrient signaling activity via the mTOR pathway [45,46,47]. However, the effect of BCAAs on muscle mass and function in LT recipients has not been investigated. The dose, the duration of supplementation, and the method of administration (EN, PN, or dietetic program) vary between studies (Figure 3). Hence, specific recommendations cannot be drawn regarding the use of BCAA after LT. BCAA supplementation could help to avoid the metabolic load of the transplanted liver itself, especially in the immediate postoperative period when oral intake is withheld. Accordingly, further studies are required to better characterize the beneficial effects and the safety of BCAA in LDLT and orthotopic liver transplantation (OLT) recipients.

#### 3.3.2. Beta-Hydroxy-Beta-Methylbutyrate (HMB) 

HMB is an active metabolite of leucine that is generally synthesized in the muscle and the liver [48]. Only nearly 5% of leucine is converted to HMB, which results in the production of 0.2–0.4 g of HMB per day in a person weighing 70 kg [49]. HMB is widely used as a nutritional supplement in athletes to maintain skeletal muscle mass [50]. It is also used to preserve or increase lean body mass in healthy people and in patients with chronic diseases associated with muscle wasting as well as in individuals ≥ 65 y of age [51,52]. In addition, HMB supplementation appears to decrease mortality, and improve nutritional status and muscle function in the elderly compared to placebo or standard care [53,54,55,56]. The dose of HMB provided is typically 3 g/day and is considered by most researchers as the optimal dosage [49].

##### Effect of HMB on Sarcopenia after LT

The effect of HMB on muscle mass and function after LT was evaluated in two studies [51,57] (Table 2). In a prospective pilot randomized controlled study, administration of 3 g of HMB enriched formula for 30 days after LDLT significantly improved muscle function, assessed by handgrip strength (HGS) test, and increased muscle mass, assessed by skeletal muscle index (SMI) using computed tomography scan (CT scan), the reference method to assess muscle mass [51]. Similarly, in another pilot randomized controlled study, 3 g of HMB supplementation dissolved in fruit juice for 12 weeks was able to induce a statistically significant improvement in muscle mass measured by appendix skeletal muscle mass index (ASMI, by dual-energy X-ray absorptiometry (DEXA)) and mid-arm muscle circumference (MAMC) in LT male recipients [57]. In addition, muscle strength (HGS test) improved significantly in the HMB group while physical performance (6 min walk test; 6MWT and timed up and go test; TUG) did not show significant changes after the intervention [57]. Only one study evaluated the effect of 3 g of HMB on prealbumin which was ineffective [51].

##### Effect of HMB on Infections, Hospital Length of Stay and Mortality after LT 

The incidence of ACR and hospital LOS has been shown to be significantly lower after HMB administration compared to controls in one study [51]. However, the incidence of infection was not different between the groups [51]. Both studies reported that supplementation with 3 g of HMB after LT appears to be well tolerated [51,57]. Diarrhea was the most common side effect and occurred in four patients (33.3%) in the HMB group [51] (Table 2).

HMB (3 g) supplementation after LT improved muscle function and increased muscle mass in both studies included in this review [51,57]. However, muscle mass was not measured with the same tools which limit the comparison. In addition, the two studies started HMB supplementation at two different times after LT. Kamo et al. started the supplementation the day after LDLT and lasted for 30 days [51] while Lattanzi et al. started the supplementation 30 days after LT and lasted for 12 weeks [57]. The effect of HMB on postoperative hospital LOS and ACR, as well as its safety, should be validated in larger studies including more females. Furthermore, evaluating the effect of HMB on nutritional status using validated tools is warranted since sarcopenia and malnutrition are interrelated.

#### 3.3.3. Immuno-Modulating Substances 

Nutrients including glutamine, ω-3 fatty acids, arginine and ribonucleic acids have been shown to display immunostimulatory effects, providing protection against microbial pathogens by enhancing immune response [58,59]. Evaluating the effect of immuno-modulating nutrients after LT could be relevant since LT recipients are at risk of infection due mainly to immunosuppressive medications they are receiving after the surgery [60]. In addition, malnutrition and decreased muscle mass, which are both frequent after LT, increase the risk of infections [61,62]. The most frequent infections after LT are pneumonia, cholangitis, and bacteremia which could lead to in-hospital death during recovery [63].

##### Effect of Immuno-Modulating Substances on Nutritional Parameters after LT 

Three studies have evaluated the effect of IMD with hydrolyzed whey peptide (HWP) on nutritional parameters and demonstrated no effect on prealbumin, zinc, BCAA, BTR, and total lymphocyte count [61,64,65] (Table 3). However, Zhu et al. showed that ω-3 fatty acids supplementation, in addition to routine treatment and BCAA, was able to improve nutritional status assessed by the prognostic nutrition index [66]. Moreover, Qiu et al. showed that patients receiving glutamine had a significant increase in the prognostic nutrition index [67].

##### Effect of Immuno-Modulating Substances on Sarcopenia after LT

Only one study assessed the effect of IMD + HWP on sarcopenia and did not find any significant difference. Sarcopenia was assessed using SMI at L3 by CT scan [65] (Table 3).

###### Effect of Immuno-Modulating Substances on Infections, Hospital Length of Stay, Acute Cellular Rejection, and Mortality after LT

The effect of IMD on infections after LT was evaluated in six studies [61,64,65,66,68,69] (Table 3). The first three studies were conducted by Kaido et al. using IMD + HWP after LDLT [61,64,65]. The first prospective pilot study showed that the incidence of post-transplant bacteremia was significantly lower in IMD + HWP compared to the control group [64]. The second study, a retrospective design including 76 LDLT, demonstrated that the incidence of bacteremia and mortality due to infection was significantly lower in the IMD + HWP group compared to the control one [61]. The third study, a retrospective study including 279 LDLT, showed that the incidence of bacteremia was significantly lower in the group receiving IMD + HWP compared to the control group [65]. Of note, all patients in the intervention and control groups included in these three studies received synbiotics after the surgery until discharge from the hospital [65]. Similarly, Zhu et al. found, in two studies, that supplementation with ω-3 fatty acids in addition to routine treatment and diet, and BCAAs for 7 days, significantly decreased the incidence of infections in OLT recipients [66,69]. These results could be explained by the anti-inflammatory and immunomodulatory role of ω-3 fatty acids which modulate the synthesis of eicosanoids [59]. However, EN enriched with glutamine, arginine, ω-3 fatty acids, and nucleic acid had no effect on the incidence of infections in another study [70]. 

**Table 3:** Nutritional intervention with immuno-modulating diet (IMD) after liver transplant (7 articles).

Authors	Study Design	Setting	Population	Intervention	Control	Duration	Results	Quality Score (MMAT)
Kamo et al., 2018 [65]	Retrospective study	Japan	279 LDLT recipients Age (y, ranges): IMD-HWP: group: 54 (18–69) Control: group 55 (18–68) M/F: 137/142	IMD-HWP group (*n* = 164): 1 kcal/mL of IMD-HWP (MEIN^®^), per 100 kcal: protein 5.0 g (main components: glutamic acid 1.09 g, aspartic acid 0.49 g, leucine 0.48 g, arginine 0.15 g), carbohydrate 13.3 g, and lipids 2.8 g, parenterally or enterally + synbiotics. The average amounts of arginine, ω-3, and glutamine given to the patients in the IMD-HWP group were 0.021, 0.024, and 0.154 g/kg/day, respectively.	Control group (*n* = 115): Conventional diet (Elental^®^), per 100 kcal: protein 4.4 g (main components: L-glutamine 0.644 g, L- serine 0.386 g, L-arginine hydrochloride 0.375 g), carbo- hydrates 21.1 g, and lipids 0.17, parenterally or enterally + synbiotics.	Started 24 h after the surgery and stopped when the patients could tolerate adequate oral intake, usually between 10 and 14 days after LDLT.	**Muscle mass:** SMI at L3 (by CT scan) did not change after IMD-HWP intervention. **Nutritional parameters:** No difference between the groups in BCAA, total lymphocyte count and zinc except for the level of prealbumin at the third week of intervention. **Infections:** The incidence of bacteremia was significantly lower in the IMD-HWP group than in the control group. **Rejection:** The incidence of ACR did not differ between the groups.	100% (very high quality)
Zhu et al., 2013 [66]	Prospective randomized, controlled clinical trial	China	98 OLT recipients Age (y, SD): PN group: 48.62 ± 14.61 PUFA group: 51.52 ± 12.41 Control group: 50.63 ± 11.73 M/F: 66/32	ω-3 fatty acids group (*n* = 33): PN supplemented with ω-3 fatty acids in addition to routine treatment. PN group (*n* = 33): PN in addition to routine treatment. Patients in the PN and ω-3 groups received BCAA (1.0 g amino acids/kg/d).	Control group (*n* = 32): Routine treatment and oral diet without additional nutrition support therapy.	7 days starting the second day after surgery.	**Nutritional parameters:** Prognostic nutrition index significantly increased only in the ω-3 group. There was significant elevation of serum albumin, prealbumin and transferrin in the ω-3 and PN groups but not in the control group. **Infections:** The incidence of infectious morbidities decreased significantly in ω-3 group. **Hospital LOS:** Hospital LOS decreased significantly in the two treated groups compared with the control group. **Rejection:** No signs of acute rejection were observed in any of the three groups. **Mortality:** Only one patient in the ω-3 group (1/33) died compared to four patients in the control (4/32) group and three patients in the PN group (3/33).	80% (high quality)
Zhu et al., 2012 [69]	Prospective randomized controlled trial	China	66 OLT recipients Age (y, ranges): PN group: 48.62 ± 14.61 PUFA group: 51.52 ± 12.41 M/F: 45/21	ω-3 fatty acids (*n* = 33): PN + ω-3 fish oil. The ω-3 fish oil was adjusted according to the weight of each patient. +BCAA (1.0 g amino acid/kg per day). PN: Nitrogen intake was 0.16 g/kg body weight per day, caloric intake was 104.5 kJ/kg per day, and lipid intake was 1.0 g/kg per day.	PN group (*n* = 33): PN without supplementation of ω-3 fatty acids. +BCAA (1.0 g amino acid/kg per day).	7 days starting the second day after surgery.	**Infections:** Infectious complications were significantly lower in ω-3 fatty acids group compared to PN group. **Hospital LOS:** The hospital LOS was significantly shortened in ω-3 fatty acids group compared with PN group. **Rejection:** No acute or chronic rejection were found in the two groups. **Mortality:** No in hospital mortality was found in the two groups. The 1-year mortality in PN group was 9.1% and in ω-3 fatty acids group was 3.1% (non-significant difference).	80% (high quality)
Kaido et al., 2012 [61]	Retrospective study	Japan	76 LDLT recipients Age (y, SD): IMD-HWP: 47.8 ± 14.8 Control group: 53.2 ± 13.4 M/F: 33/43	IMD-HWP group (*n* = 44): Same methods of Kamo et al., 2018 [65].	Control group (*n* = 36): Same methods of Kamo et al., 2018 [65].	Started 24 h after the surgery and it was stopped when the patients could tolerate adequate oral intake, usually between 10 and 14 days after LDLT.	**Nutritional parameters:** No difference between the groups in prealbumin, zinc, BCAA and total lymphocyte count. **Infections:** The incidence of bacteremia was significantly lower in the HWP-IMD group compared to the control group. **Rejection:** The incidence of ACR was similar between the groups. **Mortality:** The in-hospital mortality due to infection was not statistically different between the groups.	60% (mid-high quality)
De-fang et al., 2011 [70]	Prospective randomized controlled study	China	84 OLT recipients Age (y, SD): IMD group: 31.5 ± 14.4 Control group: 31.9 ± 17.4 M/F: 67/17	IMD group (*n* = 42): EN (Supportan^®^) containing glutamine, arginine, ω-3 fatty acid, nucleic acid, dietary fiber, and vitamin A, C, E.	Control group (*n* = 42): Regular EN (Fresubin^®^) containing protein, molybdenum, adipose, Selenium and carbohydrate.	Started 12 h after LT and stopped until patients could acquire the food corresponding to more than 50% of energy requirement, and then kept on taking orally 400 mL/d Supportan^®^ or 500 mL/d Fresubin^®^, which lasted at least until the ^7t^h day after LT.	**Infections:** No difference in the incidence of infections in the two groups. **Rejection:** There was no occurrence of immunological rejection in both groups. **Safety of the intervention:** There was no occurrence of side effects in the two groups.	80% (high quality)
Kaido et al. 2010 [64]	Prospective, non-randomized pilot study	Japan	30 LDLT recipients Age (y, SD): IMD-HWP: 45.7 ± 13.6 Control group: 56.2 ± 10.2 M/F: 13/17	IMD-HWP group (*n* = 10): Same methods of Kamo et al., 2018 [65].	Control group (*n* = 20): Same methods of Kamo et al., 2018 [65].	Started 24 h after the surgery and it was stopped when the patients could tolerate adequate oral intake, usually between 10 and 14 days after LDLT.	**Infections:** The incidence of post-transplant bacteremia was significantly higher in the control group compared with the IMD-HWP group. **Hospital LOS:** The hospital LOS in the IMD-HWP group was significantly shorter than in the control group. **Rejection:** The incidence of the ACR was similar between the groups.	60% (mid-high quality)
Qiu et al., 2009 [67]	Prospective randomized study	China	65 OLT recipients Age (y, SD): TPN group: 45.92 ± 11.82 Gln group: 50.75 ± 10.47 Control group: 48.12 ± 12.89 M/F: 40/25	TPN group (*n* = 22): total PN without glutamine in addition to their routine treatment. Gln group (*n* = 22): Total PN with alanylglutamine (Ala-Gln) in addition to their routine treatment.	Control group (*n* = 21): Routine treatment without additional nutritional support therapy.	7 days starting from the second day after the surgery.	**Nutritional parameters:** Prognosis nutritional index, albumin, prealbumin, and transferrin were significantly higher in TPN groups compared to the control group. No significant difference in total protein was observed among these groups. **Hospital LOS:** The two TPN groups showed a significant decrease in hospital LOS compared with the control group. **Rejection:** No signs of ACR were evident in these groups. **Mortality:** No significant difference in 1- or 3-year survival rates between groups. **Safety of the intervention:** Infusion of the Ala-Gln dipeptide solution was free of side effects.	40% (mid-low quality)

ACR: acute cellular rejection; BCAA: branched-chain amino acids; CT scan: computed tomography scan; EN: enteral nutrition; F: female; HGS: handgrip strength; IMD-HWP: immuno-modulating diet with hydrolyzed whey peptide; LDLT: living donor liver transplant; LOS: length of stay; LT: liver transplant; M: male; MAMC: mid-arm muscle circumference; MMAT: mixed methods appraisal tool; OLT: orthotopic liver transplantation; PN: parenteral nutrition; SD: standard deviation; SMI: skeletal muscle index; y: years.

In a prospective pilot study, IMD + HWP significantly decreased postoperative hospital LOS compared to the control group who received standard care treatment and oral diet without additional nutrition support [64]. Similarly, hospital LOS was significantly shorter in the group receiving ω-3 fatty acids, in addition to PN and BCAA, for 7 days after LT compared to PN and BCAA group [69]. Contradictorily, in two prospective randomized studies, adding glutamine or ω-3 fatty acids to PN after LT did not exert any additional benefits on the postoperative LOS [66,67]. Additionally, adding glutamine or ω-3 fatty acids to PN had no effect on 1-year or 3-year survival rates [66,67,69]. The incidence of ACR was comparable between IMD + HWP and control groups in three studies conducted by Kaido et al. [61,64,65]. In the other four studies included, there was no sign of ACR [66,67,69,70]. It is important to note that the follow-ups were completed for the whole duration of the studies (between 7 and 14 days), no longer follow-up was performed. Only two studies reported that nutrition enriched with glutamine, arginine, ω-3 fatty acids, nucleic acid, or only glutamine was well tolerated with no reported adverse symptoms [67,70].

Nutrition enriched with immuno-modulating substances reduced infections after LT. ESPEN guidelines recommend an immuno-modulating formula (enriched with arginine, ω-3 fatty acids, and nucleotides), especially for patients with marked severe nutritional risk after a major surgery [9]. However, their effects on malnutrition, sarcopenia, hospital LOS, ACR, and mortality are inconclusive. This lack of consensus could be explained by the variety of experimental designs including different compositions of IMD and the type of administration (with HWP, BCAA, or PN). It remains uncertain whether future studies should focus on the dietary combination with mixed substances or using a single-substance approach. Further additional large and well-constructed RCTs need to be conducted to ensure more robust conclusions.

##### 3.3.4. Enteral Nutrition

Early EN and PN after LT both proved to be equally effective strategies with regard to the maintenance of adequate nutritional status [71]. However, EN is a safer alternative to PN and has the potential advantage of maintaining intestinal trophism more effectively [72]. It was reported that EN could stimulate bile flow and portal blood flow, prevent intestinal mucosal atrophy, and preserve intestinal structure and functions [73]. This effect may help prevent bacterial translocation and enteric-origin infections [66].

###### Effect of Enteral Nutrition on Nutritional Parameters after LT 

Three studies evaluated the effect of EN on nutritional parameters compared to PN [71,74,75] (Table 4). Hasse et al. showed that patients who were tube-fed had an overall greater cumulative intake of calories and protein and a quicker nitrogen balance recovery compared to the control group receiving PN [74]. However, Wicks et al. concluded that starting EN 18 h after OLT was comparable to PN [71]. In this study, nutritional parameters such as MAMC, triceps skinfold thickness (TSF), and biceps skinfold thickness did not change in both groups [71]. Similarly, Kim et al. showed that nutritional parameters such as body mass index (BMI), mid-arm circumference (MAC), TSF, subjective global assessment (SGA), and MAMC did not differ between EN and PN groups [75].

###### Effect of Enteral Nutrition on Infections after LT 

Five studies evaluated the effects of EN on infections after LT [46,71,74,75,76] (Table 4). In a prospective randomized controlled pilot study, the incidence of bacterial infection was significantly lower in the EN group compared to the PN group [75]. In a retrospective study, early EN within the first 48 h was performed for 24% of LDLT recipients, on a case-by-case basis, between 2003 and 2007 and within the first 24 h after the surgery for all LDLT recipients between 2008 and 2011 [46]. The incidence of postoperative sepsis was significantly lower in the 2008–2011 group compared to 2003–2007 [46]. In another retrospective study that included 346 LDLT recipients, starting EN nutrition as early as 12 h after LT was associated with a lower rate of infections compared to no medical nutrition support [76]. The incidence of bacterial sepsis was 8-fold higher in patients without early EN within 48 h after operation [76]. In a prospective randomized study including 50 LDLT, the incidence of viral infections was significantly lower in the EN group compared to the control group [74]. No statistically significant differences were noted between the two groups regarding bacterial and fungal infections [74]. Contradictorily, another study revealed no difference in the incidence of infections between EN and PN groups [71].

###### Effect of Enteral Nutrition on Hospital Length of Stay, Acute Cellular Rejection, and Mortality after LT 

Mortality, ICU stay, hospital LOS and ACR did not differ between EN and PN groups in three studies [71,74,75] (Table 4). All the studies started EN between 12 and 48 h after LT and ceased it when oral diets were initiated or when oral intake reached 50% to 75% of requirements from a regular diet (Figure 3). Therefore, the duration of interventions was variable between studies and even between patients of the same study. The safety of EN was reported in three studies [71,74,75]. Overall EN was well tolerated. In the study of Kim et al., two patients (12%) could not tolerate early enteral feeding, one had ileus and the other had vomiting [75]. The remaining 15 patients who received early enteral feeding tolerated it well. Hasse et al. reported that four patients complained of irritation from the feeding tube, and two patients had single occurrences of vomiting [74]. Finally, Wicks et al. reported that EN was well tolerated after LT [71].

Based on these four studies included in this review, EN nutrition reduces the risk of infection after LT. The effect of EN on nutritional parameters should be validated using adequate tools. No study has assessed the effects of EN on sarcopenia. Currently, ESPEN recommends to start early EN (12 h) together with selected probiotics after LT to reduce infection rates [77]. When EN is impossible or not practicable, PN should be preferred to no feeding [77].

##### 3.3.5. Probiotics and Prebiotics

Bacterial infection is the most common cause of morbidity in the first three months after LT [78]. Infections may be caused by surgical trauma after LT which affects gut microbial flora and mucosa resulting in gut barrier dysfunction and intestinal microbial imbalance which may further aggravate systemic inflammation and depress immune function [32]. LT recipients are also at risk of foodborne illness because of immunosuppressive medications that increase vulnerability to infections [79,80]. Due to the high risk of infection after LT, patients receive food safety advice including thorough cleaning, prevention of cross-contamination, and maintaining safe temperatures during cooking [80]. 

Probiotics are bacteria that can provide beneficial effects on the gut microbial flora by maintaining the balance of resident bacteria in the bowel [81,82]. Probiotics can stabilize the intestinal barrier by stimulating epithelial growth, mucus secretion, and motility as well as enhance innate immunity [83]. Furthermore, the administration of probiotics suppresses the growth of potentially pathogenic microorganisms [83]. By contrast, prebiotics are ingredients made from food that can stimulate the proliferation of probiotics [84]. Prebiotics have been suggested to reduce translocation by stimulation of commensal microflora growth and subsequent increase in the production of short-chain fatty acids, which are known to stabilize the intestinal barrier and the local immune system [85]. Synbiotic is a product that contains both probiotics and prebiotics and where the prebiotic compound selectively favors the probiotic compound [86].

###### Effect of Probiotics and Prebiotics on Nutritional Parameters after LT 

Only two studies evaluated the effect of synbiotics on nutritional parameters. In both studies, nutritional parameters such as transferrin and BMI did not differ significantly throughout the groups after supplementation with a mixture of probiotics and fibers or lactic acid bacteria and fibers [87,88].

###### Effect of Probiotics and Prebiotics on Infections after LT 

The effect of probiotics, prebiotics, or synbiotics on infection after LT was studied in four RCTs [84,87,88,89,90] (Table 5). Mallick et al. studied the effect of synbiotics containing *Lactobacillus acidophilus, Bifidobacterium longum, Bifidobacterium bifidum,* and *Bifidobacterium lactis* as probiotics and fructooligosaccharide inulin as a prebiotic for 2 weeks after LT [89]. These synbiotics reduced significantly bloodstream infections in comparison to the placebo [89]. Similarly, Zhang et al. found that early EN supplemented with synbiotics significantly reduced the incidence of bacterial infections following LT compared to only prebiotics. In addition, in the study of Eguchi et al., synbiotic therapy including three different types of bacteria (*Bifidobacterium breve, Lactobacillus casei,* and *galactooligosaccharides*) decreased the rate of infections in the intervention group compared to the control group [84]. The same results were seen in two studies by Rayes et al. using two different interventions [87,90]. In the first study, participants received a composition of four lactic acid bacteria (probiotics) and four fibers (prebiotics) [87]. The incidence of postoperative bacterial infections was significantly reduced in the group with lactic acid bacteria and fibers compared to the group with only fibers [87]. In the second study, postoperative sepsis and wound infection rates were significantly lower in the group that received living probiotics compared to the groups with inactivated *lactobacilli* and selective bowel decontamination [90].

The mean duration of antibiotic therapy was significantly shorter in the studies of Zhang et al. and Rayes et al., 2005, as well as, in the study of Rayes et al., 2002 but the difference did not reach statistical significance [87,88,90]. The incidence of ACR was lower after the use of four lactic acid bacteria and four fibers compared to the control group [87]. Likewise, the hospital LOS in the ICU and mortality did not significantly differ after prebiotics or probiotics administration [84,87,90]. Side effects were investigated in three studies; both probiotics and fibers as well as enteral formula and Lactobacillus were well tolerated after LT [87,88,90] (Table 5).

Based on these four prospective studies, combined fibers and probiotics could lower the incidence of bacterial infections and shorten the duration of antibiotic therapy following LT. ESPEN recommends, in their recently published practical guidelines, to start early EN together with selected probiotics after LT to reduce infection rates [77]. However, the length of administration, type (prebiotics, probiotics, or synbiotics) as well as the dose and type of probiotics used must be clarified. In contrast to antibiotics, combined fibers and probiotics are relatively cheap and do not cause resistant strains or serious side effects [88].

##### 3.3.6. Vitamin D

Vitamin D, a fat-soluble vitamin, plays an important role in bone metabolism, regulates gene expression in multiple tissues, and increases calcium intestinal absorption [91]. Vitamin D deficiency is present in 91% of LT recipients [92]. While LT has been reported to improve serum vitamin D concentrations, glucocorticoid therapy in the three to six months post-LT could result in vitamin D deficiency [93]. Malnutrition is one of the main causes of low vitamin D in LT recipients. Accordingly, vitamin D requirements seem to be especially high in the context of LT and vitamin D supplementation is recommended [93].

###### Effect of Vitamin D on Infections and Acute Cellular Rejection after LT

Recently, vitamin D has been drawing attention due to its role in sarcopenia. Several studies have shown that serum level of vitamin D is independently related to muscle mass loss and muscle strength decline in older people [94,95] whereas vitamin D supplementation is effective to increase muscle strength in the same population [96]. The beneficial effect of vitamin D on muscle mass and function could be explained by four mechanisms: (1) mediation of vitamin D receptor (VDR) expression in skeletal muscle; (2) suppression of the activity of atrophy-related transcription factors; (3) stimulation of protein synthesis via mTORC1, hence signaling the induction of skeletal muscle hypertrophy and; (4) effects on the function of mitochondrial oxidative phosphorylation in the skeletal muscle [97]. 

Despite these potential beneficial effects, according to our research, no intervention study has evaluated the effect of vitamin D muscle mass and function in LT recipients nor on nutritional parameters. Previous reports show that vitamin D has a protective effect against rejection and infection in the clinical settings of solid organ transplantation, such as kidney and lung [98,99]. Doi et al. showed, in a retrospective study including LDLT and OLT recipients, that vitamin D deficiency in the post-transplant period was associated with lower survival after the surgery. However, post-LT vitamin D supplementation did not influence overall survival [91]. On the contrary, Zhou et al. found that the vitamin D supplementation group displayed lower mortality 18 months post-transplantation compared to the group without vitamin D supplementation [100]. Vitamin D supplementation decreased the incidence of ACR after LT in the three studies included in this review [91,100,101]. In addition, the incidence of infections was significantly higher in the non-supplemented group compared with the vitamin D-supplemented group [100] (Table 6).

Based on these results, vitamin D supplementation should be considered after LT, especially to reduce the incidence of ACR. However, the clinical effects of vitamin D supplementation on other LT outcomes remain unclear. Intervention studies including vitamin D administration post-LT have used different doses and different forms of vitamin D. Grant et al. published a protocol aiming to optimize vitamin D levels post-LT by giving 2500 units/day of vitamin D for 12 weeks after LT [92]. Using this intervention, 78% of patients reached minimum guideline levels (30 ng/mL). Future RCTs post-LT should be performed to confirm vitamin D’s beneficial effects post-LT on ACR and to investigate its effect on sarcopenia. Currently, vitamin D supplementation is recommended after LT since it is associated with a lower risk of ACR [92,93]. 

### 3.4. Tools to Assess Nutrition Risk, Nutritional Status and Sarcopenia

Among the common limitations of the studies included in this review, in addition to nutritional risk not being screened, nutritional status was not assessed using validated tools, thus limiting the interpretation of the results. Given the different existing tools to screen or diagnose malnutrition and sarcopenia, proper validation should be completed and tested in future studies.

#### 3.4.1. Screening for Malnutrition

Malnutrition can be screened by several validated tools, usable by untrained healthcare professionals in order to optimize the chances of efficiently identifying patients at risk of malnutrition [102]. These include Simple Screening Tools (#1 and #2), Malnutrition Screening Tool (MST), Mini Nutritional Assessment-Short Form (MNA-SF), Nutritional Risk Screening 2002 (NRS-2002), Short Nutritional Assessment Questionnaire (SNAQ), Canadian Nutrition Screening Tool (CNST) or the Malnutrition Universal Screening Tool (MUST); the latter being recommended by the ESPEN [103,104]. The CNST has been validated and tested for reliability in Canadian hospitals and is now recommended by the Canadian Malnutrition Task Force for malnutrition screening [105]. NRS- 2002 and MUST were tested after LT and they both have a sensitivity higher than 80% to predict deaths after LT and a specificity higher than 60% [104]. Further validation is needed for all the other tools with respect to clinical outcomes after LT.

#### 3.4.2. Diagnosis of Malnutrition 

Patients identified at risk of malnutrition using screening tools require a diagnosis to confirm malnutrition in terms of presence and severity. Nutritional status evaluation is the first step to addressing adequate nutritional therapy [106]. A detailed nutritional assessment by a registered dietitian should then be performed. The SGA, a simple bedside method, has been widely used as a validated method to diagnose malnutrition. SGA combines patient-based information (weight loss, dietary intake, gastrointestinal symptoms, functional status) and physician-based entities (nutrition requirements, muscle waste, fat stores, edema) [107,108]. However, SGA has several limitations including that its accuracy depends on the evaluator’s experience. The Royal Free Hospital Global Assessment (RFH-GA) is another tool to diagnose malnutrition. The RFH-GA combines physical markers BMI, TSF, MAMC, and patient-reported dietary intake [109]. Both SGA and RFH-GA may be affected by fluid retention which is however often resolved following LT. 

As sarcopenia is often associated with malnutrition, assessing body composition (muscle and fat mass) is an interesting and objective method to confirm malnutrition [110].

#### 3.4.3. Screening of Sarcopenia 

As for malnutrition, sarcopenia screening should be a rapid and simple process. Probable sarcopenia or pre-sarcopenia is present when low muscle strength is detected without any change in muscle mass or quality [17].

The SARC-F, a simple questionnaire made up of 5 items [111], has a very high specificity but low sensitivity in predicting low muscle strength [112]. Combination of SARC-F with calf circumference has been proposed to improve its sensitivity [113]. However, in addition to inter-measurer variability, there is controversy in the literature regarding the latter [112]. Other screening tools for sarcopenia screening include a screening grid for low muscle mass by age and BMI and a screening formula to identify older adults at high risk for sarcopenia based on gender, age, HGS, and calf circumference [114]. Major limitations of these tools include the validation only in patients aged 65 years and above for the former and specificity to the Japanese population for the latter [115].

#### 3.4.4. Screening of Sarcopenic Obesity 

Sarcopenic obesity (SO), defined as the simultaneous presence of sarcopenia and obesity, which may be difficult to diagnose clinically [116], is associated with increased risk of morbidity and mortality [106]. 

In their recently published consensus statement, the ESPEN and the European Association for the Study of Obesity (EASO) recommend for the screening of SO the detection of the presence of an elevated BMI or waist circumference with ethnicity specific cut points and surrogate indicators of sarcopenia (e.g., clinical symptoms, risk factors) or validated questionnaires (e.g., SARC-F) [116].

#### 3.4.5. Diagnosis of Sarcopenia

After identifying patients at risk of sarcopenia, a full evaluation of sarcopenia should be performed. As mentioned, sarcopenia definition includes three components: muscle strength, muscle quantity, and muscle quality [17]. Sarcopenia diagnosis is confirmed when, in addition to low muscle strength, low muscle quantity or quality is present whereas severe sarcopenia is defined by low muscle strength, low muscle quality/quantity, and low physical performance [17]. 

Muscle strength should be evaluated with the use of HGS as it is simple, cost-effective, and may be repeatedly measured [17]. Muscle mass may be assessed using different techniques, such as MAMC, DEXA, bioelectrical impedance analysis (BIA), muscle ultrasound, Magnetic Resonance Imaging (MRI), and CT scan [117].

The latter is the reference method to evaluate muscle mass, with particular focus on the SMI at the level of the 3^rd^ lumbar vertebra using sex-specific cut-offs (<50 cm^2^/m^2^ for men and <39 cm^2^/m^2^ for women) [118]. SMI has shown a strong correlation with clinical results and a good correlation with whole body muscle mass [119] whereas lower SMI correlates with higher wait-list mortality [18]. However, it is important to note that, in clinical practice, a CT scan is rarely used as it is expensive and exposes patients to radiation [120]. Muscle ultrasound was shown to have good validity to estimate muscle mass when compared to CT scan but many concerns remain regarding the expertise of operators, interpretation in the presence of fluid overload, or limitation by poor echogenicity [121]. In a prospective study, combining BMI and thigh muscle thickness (measured by ultrasound) was able to identify sarcopenia in patients with cirrhosis and correlated with cross-sectional imaging values (either CT scan or MRI) [122].

Muscle quality refers both to micro-and macroscopic changes in muscle architecture and composition and to muscle function delivered per unit of muscle mass [123]. Highly sensitive imaging tools such as MRI and CT have been used to assess muscle quality in research settings, by determining the infiltration of fat into muscle [17]. Myosteatosis is a pathological fat accumulation in skeletal muscle and it is radiologically identified as attenuated mean skeletal muscle radiodensity (HU) on CT scan [124]. These measures may provide additional insight into possible relationships and mechanisms of poor muscle function and health but require validation before routine use in clinical settings [102]. In the future, assessments of muscle quality are expected to help guide treatment choices and monitor response to the treatment [17].

Physical performance is assessed using the chair-stand test (CST), gait speed, short physical performance battery (SPPB) (combination of CST, gait speed, and a balance test), TUG test, and 6MWT [17]. The EWGSOP diagnostic criteria consider chair-stand time as a discrete measure of muscle strength, with a time over 15 s indicative of low strength [17].

#### 3.4.6. Diagnosis of Sarcopenic Obesity

Diagnosis to confirm or reject SO should always follow a positive screening result. The diagnosis of SO should initially include an assessment of skeletal muscle function (e.g., HGS, knee extensor strength, or CST) followed by an assessment of body composition (e.g., DEXA, BIA, or CT scan) where the presence of excess adiposity and low skeletal muscle mass or related body compartments confirm the diagnosis of SO [116]. 

## 4. Discussion and Future Considerations

The main aim of this narrative review was to assess the impact of nutritional interventions on malnutrition and sarcopenia after LT. Articles included in this review used different tools (food intake, BTR, BCAA to AAA ratio, retinol-binding protein, protein and calorie intake, energy metabolism, prognostic nutrition index, serum albumin, prealbumin, transferrin, body weight, total body fat, and total body protein) as nutritional parameters. It is important to mention that serum albumin and prealbumin are not components of malnutrition definition, since it is rather inflammation that leads to lower serum concentrations of albumin and prealbumin [125].

In this narrative review, 25 articles using different nutritional interventions after LT were identified: three studies using BCAA, two studies with HMB, seven studies looking at IMD, five studies using EN, five studies including prebiotics and/or probiotics, and three studies addressing vitamin D (Table 1, Table 2, Table 3, Table 4, Table 5 and Table 6). Among the 25 studies included, 14 assessed at least one parameter associated with nutritional status, most of them using biochemical data and six studies found beneficial effects of the intervention on nutritional parameters such as the amount of food intake, BTR, retinol-binding protein, BCAA to AAA ratio, nitrogen balance, prognostic nutrition index, and transferrin [39,40,41,67,69,74]. In these six studies with a beneficial effect on nutritional parameters, three used BCAAs, two used IMD, and one used EN [39,40,41,67,69,74]. All the studies using BCAAs improved at least one parameter associated with nutritional status [39,40,41]. However, the dose, the duration of supplementation, and the method of administration vary between studies (Figure 3). Regarding the two studies using IMD, they used varied experimental designs and especially different compositions of IMD (ω-3 fatty acids or glutamine) [66,67]. Similarly, nutritional status was not measured using validated tools in these three studies. To validate these results, future studies should measure the effect of BCAAs, IMD, and EN on nutritional status using validated tools.

Among the 25 studies included, four studies measured at least one component of sarcopenia, namely muscle mass, muscle function or muscle performance [51,57,65,74]. Only the two studies using HMB (3 g during 12 weeks) found beneficial effect on muscle mass assessed by SMI using CT scan or ASMI using DEXA [51,57]. In these studies, HMB (3 g) also improved muscle function assessed by HGS and was without effect on physical performance assessed using 6MWT and TUG test [51,57]. The duration of the intervention and the tools used were different which limits the comparison (Figure 3). Hence, future studies should aim at corroborating these findings and elucidate the optimal intervention duration.

Our review also sought to determine the effect of nutritional interventions after LT on infections, hospital LOS, ACR, and mortality. The effects of nutritional interventions on the incidence of infections have been extensively studied in 19 of the 25 included articles. Among these articles, 14 showed beneficial effect on lowering infection, using pre- or probiotics, IMD, and EN [46,61,64,65,66,74,75,76,84,87,88,89,90,100]. Based on our review, we can conclude that nutrition enriched with IMD, EN and pre- and probiotics reduced infections after LT. ESPEN recommends starting EN early after LT together with selected probiotics to reduce infection rates without specifying the type or the dose [9]. Future studies should target the optimal dose of synbiotic and the type of IMD as well as the optimal type and dose of EN to decrease the incidence rate of infection after LT. Table 7 provides a synthesis of the interventions discussed in this narrative review.

Hospital LOS was assessed in 13 studies. Our review revealed only six interventions were effective in reducing hospital LOS (four studies using IMD, one with BCAA and one with HMB) [51,61,64,66,67,75]. ACR was assessed in eleven studies with beneficial effects in only four of them; three with vitamin D and one using HMB [51,91,100,101]. Finally, only one of the 10 studies assessing mortality was effective in reducing it using vitamin D supplementation after LT [100].

The 25 articles retained in our review included LDLT and/or OLT recipients. The patient survival rate is similar between OLT and LDLT, being 92%, 84% and 76% at 1, 3 and 5 years, respectively, according to the United Network of Organ Sharing [126]. There is a contradiction in the literature regarding which type of LT is more beneficial [127,128], but certainly LDLT and OLT have different complication profiles [129]. The most important difference between OLT and LDLT is the timing of surgery [46] as the waiting time before LT for LDLT recipients is lesser compared to OLT recipients, meaning they may be less ill at the time of surgery. Importantly, prolonged waiting times may worsen outcomes after LT when patients’ nutritional status is already compromised [130]. For these reasons, the population (LDLT or OLT) must be considered when interpreting the results since OLT recipients may be sicker compared to LDLT recipients.

All the studies included in our review evaluated the effect of different interventions in the early phase (within three months) after surgery (Figure 3). There is a need for nutritional studies aiming for the long-term period after LT as other problems and needs may persist or arise. Lim et al. showed that malnutrition was still present in 26% and 11% of patients at six and 12 months after LT, respectively [104]. In the long term after LT, 50–60% of LT recipients develop metabolic syndrome due to both an increase in caloric intake and unfavorable metabolic effects of immunosuppressive drugs [131]. One of the most frequent metabolic disorders is the development of new-onset diabetes mellitus after LT (NODAT) in patients who were not diabetic before LT [132]. 

Excessive weight gain and obesity are also frequent nutritional problems in the long term after LT. Studies report a mean weight gain between 2 and 9 kg within the first year post-LT [133]. In an article by Carias et al. analyzing 207 patients, SO was detected in 13% of sarcopenic patients in pre-transplant and in 42% of patients six months after LT [134]. Changes in the body composition of transplant recipients are characterized by an early fat mass gain while muscle mass may not recover completely [135]. The excess energy intake, physical inactivity, low-grade inflammation, insulin resistance, and changes in hormonal milieu may lead to the development of SO [136], whereas immunosuppression medications may also affect body composition after LT [137].

At 2 and 3 years following transplantation, continuous weight gain contributes to new-onset obesity in 22–38% of patients [133]. Weight gain and obesity seem to be related to higher scores for specific eating behaviors of uncontrolled and emotional eating as well as cognitive restrain [138]. Understanding eating behaviors after LT could be key to acquiring knowledge about dietary intake and its impact on nutritional modifications that occur after LT. This could help to address appropriate nutritional intervention in this postoperative period.

## 5. Conclusions

Adequate nutrient intake is important for recovery after LT and for preventing complications (such as malnutrition and sarcopenia) that could persist or newly appear after the transplantation. Therefore, the absence of nutritional therapy after LT increases the risk of malnutrition and sarcopenia post-operatively. Based on this review, BCAA supplementation was effective in improving nutritional parameters after LT. Given the heterogeneity between the studies, these results need to be validated with high quality RCTs. Furthermore, HMB (3 g) was effective in improving muscle mass and function after LT. Nutritional intervention enriched with immuno-modulating substances (arginine, ω-3 fatty acids, and glutamine), EN and combined prebiotics and probiotics could reduce infections after LT. However, the lack of specific recommendation using these interventions is explained by the heterogeneity between the studies in the experimental design such as the length of administration, the composition, the dose, and the type of administration. Finally, vitamin D supplementation after LT should be considered, especially to reduce the incidence of ACR. The dose of 2500 units/day of vitamin D for 12 weeks after LT was able to improve vitamin D level and reached minimum guideline levels. Further additional high quality, multi-center RCTs are required in order to ensure robust conclusions. In addition, evaluating the effect of a nutritional intervention on nutritional status and sarcopenia using the recommended methods and tools is mandatory since both are strongly correlated morbidity and mortality after LT.

## Figures and Tables

**Table 1:** Nutritional intervention with branched-chain amino acids (BCAA) after liver transplant (3 articles).

Authors	Study design	Setting	Population	Intervention	Control	Duration	Results	Quality Score (MMAT)
Krapf et al., 2021 [39]	Prospective randomized study	Austria	57 LT recipients (*n* = 36) or major liver resection surgery (*n* = 21) Age (y, SD): 58 ± 9 M/F: 37/20	BCAA group (*n* = 24): Dietetic program composed of ingredients naturally rich in BCAA and low in AAA.	Control group (*n* = 33): Standard hospital meals.	14 days started on day 1 (±4) after the surgery.	**Nutritional parameters:** The amount of food intake measured with daily questionnaires was significantly higher in the BCAA group compared to the control group. BCAAs levels were not significantly different between the groups.	40% (mid-low quality)
Yoshida et al., 2012 [41]	Prospective randomized pilot study	Japan	25 LDLT recipients Age (y, SD): BCAA group: 52.6 ± 10.2 Control group: 48.5 ± 4.4 M/F: 11/13	BCAA group (*n* = 12): BCAA enriched nutrients; 2 packages per day of Aminoleban^®^ EN (one package contains 6.075 g of BCAAs (leucine 2.25 g, isoleucine 2.04 g, and valine 1.785 g)); orally or enterally + standard diet adjusted to 30–35 Kcal (-420 Kcal) and 1.2–1.3 g (−27 g) of protein per kg of ideal body weight per day.	Control group (*n* = 13): Standard diet adjusted to 30–35 kcal and 1.2–1.3 g of protein per kg of ideal body weight per day.	Started from 7 days to 1 day before LDLT and then restarted from 3 days to 4 weeks after LDLT.	**Nutritional parameters:** BTR and retinol binding protein significantly improved in the BCAA group compared to the control group. Energy metabolism assessed by non-protein respiratory quotient significantly increased in the BCAA group but not in the control group. **Infections:** No significant differences between the groups.	60% (mid-high quality)
Reilly et al., 1990 [40]	Prospective randomized study	USA	28 LT recipients who had hypoalbumin-emia before LT Age (y, SD): PN group: 51 ± 9 BCAA group: 44 ± 11 Control group: 50 ± 14 M/F: 13/15	BCAA group (*n* = 10): BCAA (Leucine 7.1 g/L, isoleucine 6.2 g/L, and valine 6.3 g/L) + PN (35 kcal/kg/day and 1.5 g/kg/day of protein). PN group (*n* = 8): PN (35 kcal/kg/day and 1.5 g/kg/day of protein).	Control group (*n* = 10): Without nutritional support.	7 days started on day 1 after LT.	**Nutritional parameters:** BCAA to AAA ratio was significantly higher in BCAA group compared to PN group and control group. PN with and without BCAA were able to achieve the nitrogen balance immediately after LT. **Hospital LOS:** Control group patients stayed longer in ICU without statistically significant difference. **Safety of the intervention:** BCAA were well tolerated immediately after LT.	60% (mid-high quality)

AAA: aromatic amino acids; BCAA: branched-chain amino acids; BTR: BCAA-to-tyrosine ratio; EN: enteral nutrition; F: female; LDLT: living donor liver transplant; LOS: length of stay; LT: liver transplant; M: male; MMAT: mixed methods appraisal tool; PN: parenteral nutrition; SD: standard deviation; y: years.

**Table 2:** Nutritional intervention with beta-hydroxy-beta-methylbutyrate (HMB) after liver transplant (2 articles).

Authors	Study Design	Setting	Population	Intervention	Control	Duration	Results	Quality Score (MMAT)
Kamo et al., 2020 [51]	Prospective randomized controlled pilot study	Japan	33 LDLT recipients Age (y, ranges): HMB group: 58.5 (31–66) Control group: 60 (41–63) M/F: 12/11	HMB group (*n* = 12): 3 g/day HMB-rich nutrients (2 packs per day, each contain calcium-HMB (1500 mg), l-arginine (7000 mg), and l -glutamine (7000 mg)), orally or enterally.	Control group (*n* = 11): Without intervention.	30 days starting on day 1 after LDLT.	**Muscle mass:** SMI at L3 (by CT scan) was significantly higher in the HMB group compared to the control group. **Muscle function:** HGS was significantly higher in the HMB group compared to the control group. **Nutritional parameters:** Prealbumin was not different between the groups. **Infections:** No difference between groups. **Hospital LOS:** Postoperative hospital LOS was significantly shorter in HMB group compared to the control group. **Rejection:** The incidence of ACR was significantly lower in the HMB group compared to the control group. **Safety of the intervention:** Diarrhea was the most common adverse event and occurred in four patients in the HMB group. There were no adverse events in the control group.	80% (high quality)
Lattanzi et al., 2019 [57]	Prospective randomized controlled pilot study	Italy	22 male LT recipients, 30 days after LT Age (y, SD): HMB group: 60.4 ± 5.4 Control group: 59.3 ± 7.3 M/F: 22/0	HMB group (*n* = 12): 3 g/day of HMB (1.5 g dissolved in 200 mL of fruit juice twice a day).	Control group (*n* = 10): 200 mL of fruit juice twice a day.	12 weeks starting from 30 days after LT.	**Muscle mass:** ASMI (by DEXA) increased significantly in HMB group but not in the control group. No statistically significant difference in fat free mass index and fat mass index. **Muscle mass:** MAMC significantly ameliorate in HMB group but not in the control group. **Muscle strength:** HGS increased significantly in the HMB group but not in the control group. **Physical performance:** 6MWT and TUG test did not show significant changes in both groups. **Safety of the intervention:** None of the patients reported side effects due to HMB consumption.	80% (high quality)

ACR: acute cellular rejection; ASMI: appendix skeletal muscle mass index; CT scan: computed tomography scan; DEXA: dual-energy X-ray absorptiometry; F: female; HGS: handgrip strength; HMB: beta-hydroxy-beta-methylbutyrate; LDLT: living donor liver transplant; LOS: length of stay; LT: liver transplant; M: male; MAMC: mid-arm muscle circumference; MMAT: mixed methods appraisal tool; SD: standard deviation; SMI: skeletal muscle index; y: years.

**Table 4:** Nutritional intervention with enteral nutrition (EN) after liver transplant (5 articles).

Authors	Study Design	Setting	Population	Intervention	Control	Duration	Results	Quality Score (MMAT)
Masuda et al., 2014 [46]	Retrospective study	Japan	204 LDLT recipients Age (y, SD): Patients without sarcopenia: 53.9 ± 10.5 Patients with sarcopenia: 54.8 ± 8.5 M/F: 103/101	EN group 2008–2011 (*n* = 105): EN (RACOL^®^ Liquid, 1 kcal/mL), EN has been routinely applied for all recipients within the first 24 h after LDLT. EN started at 20 mL/h for 12 h and increased by 20 mL/h every 12 h to a maximal dose of 60 mL/h.	EN group 2003–2007 (*n* = 99): EN (RACOL^®^ Liquid, 1 kcal/mL), on a case-by-case basis.	Started within 48h after LDLT and lasted until the patient could eat 50% to 75% of regular diet.	**Infection:** The incidence of postoperative sepsis was significantly lower in the EN group of 2008–2011 compared to the EN group 2003–2007.	80% (high quality)
Kim et al., 2015 [75]	Prospective randomized controlled pilot Study	South Korea	36 LDLT recipient Age (y, ranges): EN group: 52 (36–64) Control group: 52 (43–65) M/F: 33/3	EN group (*n* = 17): low residual EN diet (Mediwell RTH 5001). EN was started at 20 mL/h for 12 h and, if well tolerated, the rate was increased to 60 mL/h by postoperative 5 days.	Control group (*n* = 19): PN, maintained on intravenous fluid until oral diets were initiated.	EN started within 12 h after LDLT and it was discontinued once a patient could eat more than 50% of the provided regular diet.	**Nutritional parameters:** No statistically significant difference in BMI, MAC, TSF, SGA, and MAMC between the two groups. **Infections**: The incidence of bacterial infection was significantly lower in the EN group than in the control group. **Mortality, ICU stay and hospital LOS:** No statistically significant difference between the two groups. **Sides effects:** Two patients in the EN group could not tolerate early enteral feeding; one had ileus and the other had vomiting. The remaining 15 patients who received early enteral feeding tolerated it well.	60% (mid-high quality)
Ikegami et al., 2012 [76]	Retrospective study	Japan	346 LDLT recipients Age (y, SD) 51.5 ± 11.8 M/F: 166/180	EN group 2008–2011: Same methods of Masuda et al., 2014 [46].	EN group 2003–2007: Same methods of Masuda et al., 2014 [46].	Started within 48 h after LDLT and lasted until the patient could eat 50% to 75% of regular diet.	**Infections:** The incidence of bacterial sepsis was 8-fold higher in patients without early EN within 48 h after operation.	80% (high quality)
Hasse et al., 1995 [74]	Prospective randomized study	USA	50 OLT recipients (31 patients completed the study) Age (y, SD): Intervention: group 55.2 ± 12.4 Control group: 47.5 ± 13.7 M/F: 17/14	EN group (*n* = 25): EN (Reabilan^®^ HN) The infusion rate was started at 20 mL/h and was increased to 40 mL/h 24 h after the initiation of the tube feeding. If the patient tolerated 40 mL/h, the infusion rate was increased to 60 mL/h 12 h after the previous rate increase.	Control group (*n* = 25): PN, conventional electrolyte solutions, determined by hydration status of the patient.	Started 12 h after OLT until oral diet is initiated.	**Muscle strength:** HGS was not significant different between the two groups. **Nutritional parameters:** Protein and calorie intake were significantly higher in EN group compared with the control group, six days and 12 days after OLT. No significant differences were noted between the two groups for resting energy expenditure. **Infections:** Viral infections were significantly higher in the control patients compared with the EN group. **Sides effects:** Four patients complained of irritation from the feeding tube, and two patients had single occurrences of vomiting. **Hospital LOS and rejection:** No difference between the group during the first 21 posttransplant days.	20% (low quality)
Wicks et al., 1994 [71]	Prospective non-randomized study	England	24 OLT recipients Age (y, ranges): 46 (16–62) M/F: 10/14	EN group (*n* = 14): EN (Osmolite^®^) of 1 kcal/mL, nutritionally complete, isotonic formula. The energy distribution of the feed was 16–6% protein, 30–8% fat, and 52–6% carbohydrates.	TPN (TPN) group (*n* = 10): PN contained crystalline L-amino acids, carbohydrates, fat, vitamins, and minerals.	EN started post-operatively within 18 h. TPN started in 7 patients within 24 h but in the remaining 3 there were delays of up to 60 h. EN or TPN were stopped when oral intake reaches 70% of requirements from a normal diet (4 to 5 days post LT).	**Nutritional parameters:** MAMC, TSF, and biceps skinfold thickness did not change after intervention in both groups. **Infections and hospital LOS:** No statistically significant differences between groups. **Mortality:** No statistically significant differences between groups. Two patients died during the study, one from each group, the causes being unrelated to feeding method. **Side effects:** Nutritional support was well tolerated.	60% (mid-high quality)

EN: enteral nutrition; F: female; HGS: handgrip strength; LDLT: living donor liver transplant; M: male; MAC: mid-arm circumference; MAMC: mid-arm muscle circumference; MMAT: mixed methods appraisal tool; OLT: orthotopic liver transplantation; PN: parenteral nutrition; LOS: length of stay; SD: standard deviation; SGA: subjective global assessment; TPN: total parenteral nutrition; TSF: triceps skinfold thickness; y: years.

**Table 5:** Nutritional intervention with probiotics and prebiotics after liver transplant (5 articles).

Authors	Study Design	Setting	Population	Intervention	Control	Duration	Results	Quality Score (MMAT)
Mallick et al., 2022 [89]	Randomized, double-blinded, investigator-initiated, controlled trial	India	100 LDLT recipients Age (y, SD): Synbiotic group: 51.46 ± 7.46 Control group: 48.54 ± 9.97 M/F: 94/6	Synbiotic group (*n* = 50): Synbiotic drug Prowel^®^ containing *Lactobacillus acidophilus* (2.5 billion), *Bifidobacterium longum* (0.25 billion), *Bifidobacterium bifidum* (0.25 billion) and *Bifidobacterium lactis* (2.0 billion), and Fructooligosacccharide inulin (25 mg), 3 times a day.	Control group (*n* = 50): Placebo: Emptied Prowel^®^ capsules, 3 times a day.	Starting 2 days before LDLT and continued for 2 weeks after LDLT.	**Infections:** Blood stream infections were significantly lower in the synbiotic group compared to the control group, whereas the urinary tract and intra-abdominal infections were similar. **Hospital LOS:** Hospital LOS was comparable between groups. **Mortality:** 30-day mortality was comparable between groups.	100% (very high quality)
Zhang et al., 2013 [88]	Mixed study	Australia	67 LT recipients Age (y, SD): Intervention group: 57 ± 10 Control group: 55 ± 12 M/F: 36/31	Synbiotic group (*n* = 34): EN + capsule of symbiotic twice a day via the feeding tube or orally; each capsule contains 6 different probiotic strains and 27 billion organisms of beneficial bacteria.	Control group: (*n* = 33): EN with only fiber.	Started after LT when patient tolerant oral fluid and continued for at least 7 days post-LT.	**Nutritional parameters:** Serum prealbumin and BMI did not differ significantly throughout the groups. **Infections:** Synbiotic group had significantly fewer infections and shorter duration of antibiotic therapy compared to the control group. **Side effects:** EN with probiotics and fiber was well tolerated. In synbiotic group, 2/34 patients developed diarrhea and 3/34 patients had abdominal cramps. In the control group, 1/33 patients had signs of diarrhea and 6/33 patients had abdominal distension and cramps. All side effects disappeared under temporary reduction in the amount of EN. **Hospital LOS and mortality:** There is no significant difference in hospital LOS and mortality.	60% (mid-high quality)
Eguch et al., 2011 [84]	Prospective randomized study	Japan	50 LDLT recipients Age (y, ranges): Intervention: group 56 (33–66) Control: group 57 (25–68) M/F: 29/21	Synbiotic group (*n* = 25): Received synbiotic therapy (15 mg *Bifidobacterium breve*, 20 mg *Lactobacillus casei*, and galactooligosaccharides 15 g/d) 3 times per day.	Control group (*n* = 25): without synbiotic therapy.	2 days of preoperative and 2 weeks of postoperative	**Infections:** Synbiotic group had significantly fewer infections compared to the control group. **Hospital LOS and mortality:** No differences in the ICU period, hospital LOS and mortality rates between the groups.	60% (mid-high quality)
Rayes et al., 2005 [87]	Prospective randomized double-blind study	Germany	66 LT recipients Age (y, SD): Intervention group: 53 ± 2 Control group 50 ± 2 M/F: 38/28	Synbiotic group (*n* = 33): EN (Stresson^®^) +synbiotic (Synbiotic 2000^®^) twice daily via the feeding tube or orally: four lactic acid bacteria (*1010 Pediacoccus pentosaceus, Leuconostoc mesenteroides, Lactobacillus paracasei ssp. paracasei and L. plantarum*) and four fibers (2.5 g of each betaglucan, inulin, pectin, and resistant starch, totally 10 g/dose, or 20 g/day). EN contains per liter 1250 kcal, 75 g protein, 145 g carbohydrates and 42 g lipids	Control group (*n* = 33): received the fibers only.	The treatment started on the day of the operation and continued during the first 14 days after the operation. The initial infusion rate was 25 mL an hour. If well tolerated, the enteral infusion rate was increased to 1 mL/kg body weight/h from postoperative day 1 and continued for at least 8 days.	**Nutritional parameters:** Prealbumin and transferrin did not significantly differ between the groups. **Infections:** The incidence of postoperative bacterial infections was significantly lower in the synbiotic group compared to the control group. The duration of antibiotic therapy was significantly shorter in the patients receiving the synbiotic combination compared to those receiving only fibers. **Side effects:** Fibers and lactic acid bacteria were well tolerated. **Hospital LOS:** Hospital LOS did not differ between the groups.	60% (mid-high quality)
Rayes et al., 2002 [90]	Prospective, randomized controlled study	Germany	95 OLT recipients Age (y, SD): Intervention group 1: 47 ± 2 Intervention group 2: 50 ± 2 Control group: 50 ± 2 M/F: 49/46	SBD Group (*n* = 32): EN + SBD (5 mL of SBD containing 80 mg of tobramycin, 500 mg of amphotericin B, and 100 mg colistin sulfate was given orally four times a day for 6 weeks postoperatively). EN provides 1000 kcal/L, 38 g protein/L, 138 g carbohydrate/L, and 34 g lipid/L. Living Lactobacillus Group 2 (*n* = 31): EN + living Lactobacillus and one fiber. EN provides 1000 kcal/L, 40 g protein/L, 123 g carbohydrate/L, and 29 g lipid/L.	Control group (*n* = 32): Fiber containing formula plus inactivated *lactobacilli* twice daily.	Feeding began with 25 mL/h for 24 h and when tolerated by the patient was advanced to 75 mL/h on postoperative day 3 and continued until postoperative day 12.	**Infections:** The patients who received living lactobacilli plus fiber developed significantly fewer bacterial infections than the patients with SBD. In the living Lactobacillus group, the mean duration of antibiotic therapy was shorter than in the groups with inactivated lactobacilli and fiber as well as with SBD. However, these differences did not reach statistical significance. **Hospital LOS:** Hospital LOS was also longer in SBD group compared with living Lactobacillus group and control group. However, none of these differences were statistically significant **Side effects:** Both EN and Lactobacillus were well tolerated.	60% (mid-high quality)

BMI: body mass index; EN: enteral nutrition; F: female; GS: grip strength; LDLT: living donor liver transplant; LT: liver transplant; M: male; MMAT: Mixed Methods Appraisal Tool; OLT: orthotopic liver transplantation; PN: parenteral nutrition; LOS: length of stay; SBD: selective bowel decontamination; SD: standard deviation; y: years.

**Table 6:** Nutritional intervention with vitamin D after liver transplant (3 articles).

Authors	Study Design	Setting	Population	Intervention	Control	Duration	Results	Quality Score (MMAT)
Doi et al. 2020 [91]	Retrospective observational cohort	USA	528 LT recipients (LDLT + OLT) Age (y, ranges) at the time of LT was 58 years (52–64) M/F: 350/178	N.A.	N.A.	N.A.	**Overall survival:** Regardless of the supplementation status (no supplement, pre, post, pre/post), there were no significant differences in overall survival. **Rejection:** The post-transplant supplementation of vitamin D was associated with a lower risk of ACR.	100% (very high quality)
Zhou et al., 2019 [100]	Retrospective cohort clinical study	China	141 liver allograft recipients Age at blood draw (y, SD): Intervention group 49.7(8.5) Control group 50.5(11.6) M/F: 120/21	Intervention group (*n* = 71): received oral calcitriol (250 ng/day)	Control group (*n* = 70): Non-vitamin D supplementation	1 month after LT	**Infections:** The incidence of infections were significantly higher in the non-supplementation group compared with the vitamin D supplementation group. **Rejection:** The incidence of the development of ACR was sig higher in the no vitamin D supplementation group compared with the vitamin D supplementation group. **Mortality:** Vitamin D supplementation group have lower mortality at 18 months post transplantation compared to the group without vitamin D supplementation.	100% (very high quality)
Xing et al., 2013 [101]	Prospective, randomized, controlled study	China	75 LT recipients Age (y, ranges): 48.5 (28–65) M/F: 62/13	Calcitriol group (*n* = 25): calcium gluconate + calcitriol.	Calcium only group: calcium gluconate. Control group (*n* = 25): placebo.	4 weeks after LT	Rejection: The ACR rate was significantly lower in calcitriol group compared to calcium and control group.	40% (mid-low quality)

ACR: acute cellular rejection; F: female; LDLT: living donor liver transplant; LT: liver transplant; M: male; MMAT: Mixed Methods Appraisal Tool; N.A.: non applicable; OLT: orthotopic liver transplantation.

**Table 7:** Summary of evidence on nutritional interventions after liver transplant.

Intervention	Summary of Evidence
BCAA	The effect of BCAA showed beneficial effects on markers associated with nutritional status, energy metabolism and protein turnover. The dose, the duration of supplementation, and method of administration (EN, PN or dietetic program) vary between studies. Specific recommendations cannot be drawn regarding the use of BCAA.
HMB	HMB (3 g) supplementation improved muscle function and increased muscle mass. Tools used to evaluate muscle mass and the timing of the intervention vary between studies.
IMD	IMD reduced infections. ESPEN guidelines recommend an immuno-modulating formula (enriched with arginine, ω-3 fatty acids, and nucleotides), especially for patients with marked severe nutritional risk after a major surgery. The experimental design and the type of administration (with HWP, BCAA or PN) vary between studies.
EN	EN nutrition reduces the risk of infection. ESPEN recommends to start early EN (12 h) together with selected probiotics to reduce infection rates. When EN is impossible or not practicable, PN should be preferred to no feeding.
Prebiotics and probiotics	Combined fiber and probiotics could lower the incidence of bacterial infections and shorten the duration of antibiotic therapy. ESPEN recommends starting early EN together with selected probiotics to reduce infection rates. The length of administration, type (prebiotics, probiotics or synbiotic), the dose and type of probiotics used must be clarified.
Vitamin D	Vitamin D supplementation could reduce the incidence of ACR. The dose and the form of vitamin D vary between studies.

ACR: acute cellular rejection; BCAA: branched-chain amino acids; EN: enteral nutrition; ESPEN: European Society for Parenteral and Enteral Nutrition; HMB: beta-hydroxy-beta-methylbutyrate, HWP: hydrolyzed whey peptide; IMD: immuno-modulating diet; PN: parenteral nutrition.

## Data Availability

The data that support the findings of this study are available from the corresponding author upon request.
